# Anthraquinone-2-Carboxylic Acid Is a Potential Antiviral Candidate Against Influenza Viruses In Vitro and In Vivo

**DOI:** 10.3390/v17050628

**Published:** 2025-04-27

**Authors:** Sichen Ren, Yan Luo, Huimin Tao, Ping Wang, Song Li, Jingjing Yang

**Affiliations:** 1 Sanya Research Institute of Hainan University, Hainan University, Yazhou Bay, Sanya 572000, China; rnschn@yeah.net; 2 Song Li’s Academician Workstation, School of Pharmaceutical Sciences, Hainan University, Yazhou Bay, Sanya 572000, China; 17384680560@163.com (Y.L.); wp1303544657@163.com (P.W.);; 3 School of Basic Medical Sciences, Tsinghua University, Beijing 100084, China; thm21@mails.tsinghua.edu.cn; 4 Key Laboratory of Tropical Biological Resources of Ministry of Education, School of Pharmaceutical Sciences, Hainan University, Haikou 570228, China

**Keywords:** influenza virus, A2CA, antiviral, replication, transcriptome analysis

## Abstract

Seasonal outbreaks and occasional pandemics triggered by influenza viruses annually impose considerable burdens on public health and finances. The continual evolution of viral strains with drug resistance emphasizes the urgency of discovering novel agents for influenza viruses. This study investigated a set of innovative substances derived from *Morinda officinalis* with antiviral potential against influenza virus strains. The top candidate, anthraquinone-2-carboxylic acid (A2CA), presented antiviral activity against diverse influenza virus strains, including those resistant to oseltamivir. In an influenza mouse model, the pre-administration of A2CA dose-dependently ameliorated influenza A virus (IAV)-mediated weight loss as well as protected mice from a lethal IAV infection. In addition, lung injury and cytokine dysregulation were mitigated. Further investigation revealed that IAV-induced activation of the RIG-I/STAT1 signaling pathway did not occur after A2CA treatment. A time-of-addition assay revealed that A2CA targeted the final phase of intracellular replication, which was further determined by molecular docking between A2CA and the IAV RdRp protein. Finally, transcriptome analysis revealed that the TP53TG3C, CFAP57 and SNX30-DT genes may be involved in the antiviral effects of A2CA. These results play a part in achieving a thorough comprehension of the capacity of A2CA to inhibit influenza virus infection.

## 1. Introduction

There are roughly 1 billion instances of seasonal influenza annually, with approximately 3 to 5 million of these being severe. Respiratory infection-related fatalities are in the range of 290–650 thousand [1]. The ongoing pandemic caused by the spread of influenza viruses with high pathogenicity remains a principal peril to the public. The emergence of new influenza variants via genetic mutation and re-assortment as well as increased drug resistance also raise concerns about a pandemic that can overcome human intervention strategies. Accumulating evidence indicates that a potent and disordered innate immune response predominantly drives the acute exacerbation of influenza diseases, resulting in a high mortality rate [2,3]. Currently, the treatments available in the clinic for influenza-related diseases are still limited. Moreover, few alternative agent strategies are available for treating influenza virus-infected patients who suffer from severe inflammatory responses. Therefore, novel intervention strategies are desperately needed to combat future burgeoning influenza pandemics.

*Morinda officinalis* is a member of the Rubiaceae family and is renowned for its roots (Named Bajitian in Chinese) being one of the four great southern medicines of China [4]. For centuries, Bajitian has been used as a nourishing agent for strengthening bone, invigorating the kidney and enhancing immune function when dealing with menstrual disorders, impotence, osteoporosis, dermatitis, rheumatoid arthritis as well as diabetes in China and Northeast Asia [5,6]. Based on the above facts, we hypothesized that *Morinda officinalis* has active ingredients that achieve antiviral effects through anti-inflammatory and immune-regulating functions.

**Figure 1:**
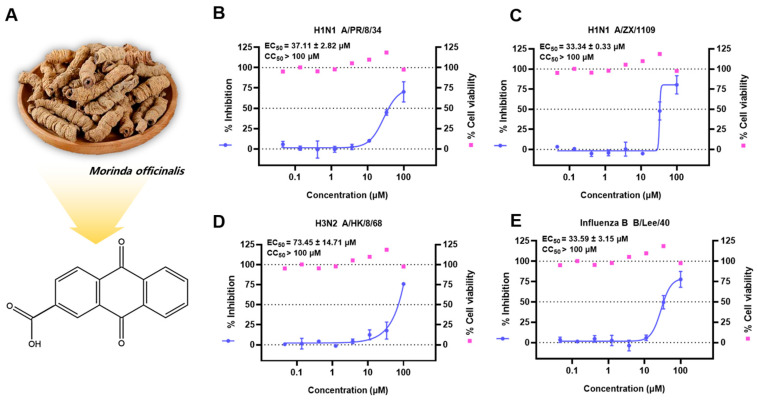
In vitro antiviral activities of A2CA derived from *Morinda officinalis* against multiple influenza virus strains. (**A**) A2CA from *Morinda officinalis*. (**B**–**E**) The protective effects of A2CA against four influenza virus strains were evaluated in MDCK cell lines using a CPE-based antiviral assay.

This study reported a set of innovative substances obtained from *Morinda officinalis* with potent anti-influenza strain capabilities. Among them, the top candidate 9,10-dihydro-9,10-dioxo-2-anthracenecarboxylic acid (anthraquinone-2-carboxylic acid, A2CA, Figure 1) is an anthraquinone compound derived from Bajitian and parts of other medicinal plants, such as *Cassia tora* (Leguminosae) seeds [7] and *Tabebuia spp*. (Bignoniaceae) inner bark (taheebo) [8]. Studies have suggested that A2CA has robust anti-inflammatory, immunoregulatory, antinociceptive and antibacterial activities [8,9,10,11]. Moreover, A2CA and its structural analogs were identified as dipeptidyl peptidase IV (DPP-IV) inhibitors, which are expected to be developed into antidiabetic drugs [12]. To comprehensively understand the antiviral mode of the top candidate A2CA, we systematically undertook a series of experiments to conduct a more detailed exploration of the effects of A2CA and the intricate mechanisms.

## 2. Materials and Methods

### 2.1. Chemicals

We reviewed all the studies in relevant databases on the chemical composition of *Morinda officinalis* and acquired 391 components (accessed 15 May 2023). A preliminary screening of the retrieved compounds was performed using Excel 2019 to remove duplicate records. Further exclusion criteria included compounds with reported anti-influenza activity, and we purchased the remaining compounds from MedChemExpress (MCE) LLC (Monmouth Junction, NJ, USA), Selleck Chemicals LLC (Houston, TX, USA) and TargetMol Chemicals Inc. (Boston, MA, USA). We ultimately obtained 13 candidate compounds (Supplemental Table S1) as well as oseltamivir acid, (-)-epigallocatechin gallate sulfate (EGCG) and favipiravir (T-705), which were used as positive controls and were supplied by MCE. All of them were made into a 100 mM solution by dissolving in DMSO and preserved at −20 °C for later use.

### 2.2. Viruses, Cells, and Animals

Influenza A/LiaoNingZhenXing/1109/2010 (H1N1 ZX/1109, oseltamivir-resistant seasonal strains) and A/Puerto Rico/8/1934 (H1N1 PR/8) strains were propagated in infected allantoic fluid of 10-day-old SPF embryonated chicken eggs at 37 °C for 48 h. Influenza A/Hongkong/8/68 (H3N2, HK/68), influenza B/Lee/40 and PR/8-NS1-GFP strains were propagated in MDCK cells in a virus growth medium, Dulbecco’s modified Eagle’s medium/Ham’s F-12 medium (DF-12) (Gibco, Grand Island, NY, USA) added with 2 μg/mL (for MDCK cells) or 0.5 μg/mL (for A549 cells) TPCK-treated trypsin (Sigma-Aldrich, St. Louis, MO, USA) and 1% antibiotics at 37 °C for 3 d. Our laboratory preserved all the virus strains, the titers of which were measured by the plaque-forming unit (PFU) assays and 50% tissue culture infectious dose (TCID_50_) assays in MDCK cells [13].

Alveolar type II-like epithelial (A549) cells, Madin-Darby canine kidney (MDCK) cells, and human embryonic kidney (HEK) 293T cells were supplied by the American Type Culture Collection (ATCC, Manassas, VA, USA). They were cultivated in a cell growth medium, Dulbecco’s modified Eagle’s medium (DMEM, Gibco, Grand Island, NY, USA) added with a 1% penicillin–streptomycin antibiotic solution and 10% fetal bovine serum (Gibco, Grand Island, NY, USA) at 37 °C under 5% CO_2_.

SPF BALB/c mice (female, aged 7 weeks, 16–18 g in weight) were purchased from Beijing Vital River Laboratory Animal Technology Co (Beijing, China). By serial dilution of the virus, we determined the dose of the H1N1 PR8 strain that was lethal to 50% of the tested mice.

### 2.3. Cytotoxicity Assay and Cytopathic Effect Inhibition Assay

We detected the antiviral activity of 13 selected components through in vitro cytopathic effect inhibition assays [14]. Briefly, we seeded 1.5 × 10^4^ MDCK cells (in 100 μL) into each well of 96-well plates. Before infecting the cells, we replaced the medium with DF-12, a specialized virus growth medium. Next, we treated the cells with varying concentrations of the test compounds or oseltamivir acid from 100 μM down to 0.005 μM at threefold dilutions. Subsequently, we introduced four different virus strains into the cells, each at an inoculation dose of TCID_50_. At 72 hpi, we measured the inhibition capacities by the CellTiter-Glo^®^ Luminescent Cell Viability Assay (Promega, Madison, WI, USA). The cytotoxicity assay was carried out in a way comparable to the above, with the only difference being medium supplementation instead of virus inoculation. Finally, we used Origin 8 software to calculate the 50% cytotoxicity concentration (CC_50_) and 50% CPE protection concentration (EC_50_).

### 2.4. Viral RNA Copy Number and Infectious Particle Inhibition Assay

Briefly, we seeded 1.5 × 10^5^ MDCK cells (in 1 mL) into each well of 12-well plates overnight. They were then untreated or treated with A2CA from 100 μM down to 1.23 μM with a threefold dilution, and inoculated with the H1N1 PR/8 strain at a 0.01 MOI. The progeny infectious particles in cell supernatants were quantified by the PFU method, and the quantity of viral RNA in cell lysates was measured in a qRT-PCR assay.

### 2.5. PFU Assays

PFU assays for viral titers were carried out as described previously [14]. In brief, we plated 1.5 × 10^5^ MDCK cells (in 1 mL) into each well of 12-well plates until a confluent monolayer formed. The samples were continuously diluted with a virus growth medium in 10-fold increments and then added to the plates. At 2 hpi, the inoculum was carefully suctioned off, and the cells were rinsed 3 times using PBS. This mixture was subsequently overlaid with a virus growth medium incorporating agarose (1%). At 72 hpi, to each plate formaldehyde (4%) was added to fix the cells for 4 h. After that, crystal violet (1%) was used to stain, and the PFU was determined.

### 2.6. Viral and Immune Curves

Briefly, we seeded 1.5 × 10^5^ A549 cells (in 1 mL) into each well of 12-well plates overnight. They were then untreated or treated with A2CA ranging at 100 μM, and inoculated with the H1N1 PR/8 strain at a 0.01 MOI. The cell lysates were collected at multiple time points (1, 3, 6, 9, 12, 24, 36, 48 or 72 hpi), and the viral RNA copy number and immune factor expression were determined by qRT–PCR assays.

### 2.7. Time-of-Addition Assay

We further conducted a time-of-addition assay as reported previously to explore the action mode of A2CA [14]. Briefly, we plated 1.5 × 10^5^ MDCK cells per well (in 1 mL) in 12-well plates overnight. Then, they were exposed to the H1N1 PR/8 strain for a period from 0 to 2 hpi. A2CA (100 μM) or T-705 (100 μM) were tested across 5 distinct time periods. To assess the viral titers, PFU assays were employed for cell supernatants, while qRT–PCR assays were utilized for cell lysates.

### 2.8. Hemagglutination Inhibition and Neuraminidase Inhibition Assays

Briefly, in a U-bottomed 96-well plate, equal volumes (50 μL) of a 4-hemagglutinating-units (4HAU) H1N1 PR/8 viral solution were blended thoroughly with series of A2CA dilutions. Then, the mixture was kept for 15 min in room temperature. Thereafter, we introduced 100 μL of fresh chicken erythrocytes (1% in PBS) to every test well. Finally, the plate was kept for at least 30 min in room temperature to allow hemagglutination. The negative control consisted of PBS only [13,14].

The neuraminidase (NA) inhibitory activity of A2CA was detected using an NA-Fluor^TM^ Influenza Neuraminidase Assay Kit (Applied Biosystems, Carlsbad, CA, USA). Oseltamivir acid was set as a positive control. The NA from the H1N1 PR/8 strain (5 × 10^4^ PFU) was applied for this assay.

### 2.9. IAV Replicon Assay

The IAV replicon assay was detected as per the previous procedures to validate the capacity of A2CA to inhibit viral replication [14,15]. Briefly, we seeded 2.5 × 10^4^ HEK 293T cells (in 100 μL) into each well of a 96-well plate overnight. The plasmids pRLSV40, polI–NS–Luc, PB1, PB2, PA and NP, which encode the luciferase reporter and influenza RdRp component, were transfected into cells by the Lipofectamine 3000 Transfection Kit (Invitrogen, Carlsbad, CA, USA). Then, A2CA and the control drugs (oseltamivir acid was negative, while T-705 was positive) were added at a concentration of 100 μM at 6 h post transfection. The Dual-Glo luciferase assay kit (Promega, Madison, WI, USA) was applied to measure luciferase activity in a microplate reader at 30 h post treatment. The curve fittings were generated by Origin 8 software.

### 2.10. Pseudotyped IAV Particles Entry Assay

We plated 1.5 × 10^4^ cells MDCK cells (in 100 μL) into each well of a 96-well plate overnight. According to the method reported previously [14,16], pseudotyped particles were assembled in HEK 293T cells. Subsequently A2CA (100 μM) and pseudotyped particles were added together to the cells. After 2 h at 37 °C, the cells underwent 3 washes with PBS and continued to be incubated for another 24 h. The Bright-Glo Luciferase Assay System (Promega, Madison, WI, USA) was used for luciferase activity determination.

### 2.11. In Vivo Antiviral Effect of A2CA

We randomly divided 30 BALB/c mice into 3 equally sized groups. A2CA at 100 mg/kg and 10 mg/kg suspended in a vehicle solution (0.5% sodium carboxymethyl cellulose) was then intragastrically (i.g.) administered to them for five consecutive days. After the third day of administration, 40 μL 3-fold the LD_50_ of the H1N1 PR/8 strain was intranasally inoculated into every mouse. The body weight and survival of each mouse were monitored daily after inoculation.

Similar experimental procedures were used for further pathological examination, influenza RNA copy number determination and infectious particle detection of lung tissues, except that the mice were sacrificed at 2 dpi. The same lung pieces were collected and weighed for each experiment, with one part of the right lobe of the lung used for the PCR assay and the other used for the PFU assay. The remaining left lungs were entirely soaked in 4% paraformaldehyde for subsequent pathological examination.

### 2.12. Quantitative Real-Time PCR

The RNA of cells and lung tissues were extracted by a TRizol reagent assay (Invitrogen, Carlsbad, CA, USA). QuantStudio^TM^ 3 and 5 Real-Time PCR Instruments (Themo fisher, Waltham, MA, USA) was then applied to carry out RT-PCR. Therein, the absolute quantification of viral RNA copy numbers was tested using a One Step PrimeScript™ RT-PCR Kit (Takara, Tokyo, Japan). Meanwhile, the relative quantification of gene expression was determined by a One Step TB Green^®^ PrimeScript™ RT-PCR Kit (Takara, Tokyo, Japan) with β-actin as an endogenous reference. The 2^−ΔΔCT^ method was applied to the obtained results. The primers and probe sequences are seen in Supplementary Table S2.

### 2.13. Fluorescence Microscopy

In brief, we seeded 1 × 10^4^ cells A549 cells (in 100 μL) into each well in a black 96-well plate overnight. The cells were rinsed 2 times with PBS prior to inoculation with the H1N1 PR/8 reporter virus at a 0.1 MOI in a virus growth medium. Then, the series concentration of A2CA was added. At 2 hpi, the cells were rinsed 2 times, and the corresponding A2CA was re-added. After 16 h, a Leica DMi8 inverted microscope was employed to capture the images. The Hoechst 33342 channel was for used nuclear visualization, whereas the GFP channel was used for virus visualization [16].

### 2.14. Western Blotting

We seeded 3 × 10^5^ cells A549 cells (in 2 mL) into each well of 6-well plates overnight. Then, the plates were incubated with the H1N1 PR/8 strain (0.01 MOI) and the indicated A2CA. At 24 hpi, the RIPA Lysis Buffer was used to extract the total protein on ice. The proteins were detected by an enhanced chemiluminescence detection system. All the antibody information can be found in Supplementary Table S3.

### 2.15. Transcriptome Sequencing and Gene Ontology (GO) Enrichment Analysis

We inoculated 5 × 10^5^ A549 cells (in 2 mL) into each well of 6-well plates overnight. Subsequently, the cells were incubated with the H1N1 PR/8 strain in the presence or absence of A2CA. After 18 h, cellular RNA was collected and subsequently subjected to Shanghai Majorbio Biopharm Biotechnology Co., Ltd (Shanghai, China). for RNA-seq analysis. High-quality RNA (1 μg) per sample was employed to make the RNA-seq transcriptome library in line with the Stranded mRNA Prep, Ligation (Illumina, San Diego, CA, USA). Sequencing was performed on a NovaSeq X Plus platform (PE150) using a NovaSeq Reagent Kit (Illumina, San Diego, CA, USA). The raw paired end reads were quality controlled using fastp with default parameters, and then clean reads were aligned to a reference genome with orientation mode by HISAT2 software (version 2.2.1). In this study, DESeq2s with *p* values < 0.05, expressed genes (DEGs) with |log2FC| ≥ 1 and *p* values < 0.05 were considered significant. Functional enrichment analysis including GO and KEGG were performed to identify the DEGs that were significantly enriched in GO terms and metabolic pathways at Bonferroni-corrected *p* values < 0.05 using [13,14].

### 2.16. Molecular Docking

We selected the IAV H1N1 RdRp protein (PDB:7NK4) and the nucleoprotein (NP, PDB: 6J1U) structure with the highest resolution in the Protein Data Bank (PDB) protein structure database (https://www.rcsb.org/) (accessed on 24 November 2024) as the docking receptor. Moreover, we selected the A2CA active molecule in the PubChem database (https://pubchem.ncbi.nlm.nih.gov/) (accessed on 24 November 2024) as the ligand. We subsequently conducted computer virtual docking prediction via the LigandFit module of Discovery Studio 2016 software. After removing water molecules and bound ligands and adding hydrogens, the possible binding sites were generated. The active ingredients were docked to the possible binding sites of multiple targets, and the affinity and docking score between the protein and the core component were used to determine the docking results. The sites with a docking score greater than 35 were selected and further analyzed using Dock Ligands (CDOCKER). The three-dimensional structure of the protein with active sites was saved in the LigandFit module, and the CDOCKER module was used to conduct computer virtual docking prediction. Finally, we used CDOCKER ENERGY to determine the binding energies and simultaneously exported 3D simulated docking schematic diagrams and 2D images to display the potential intermolecular forces and chemical bonds involved in the binding process.

### 2.17. Statistical Analysis

The results are represented as means ± standard deviation (SD). The level of statistical significance is denoted by the asterisks with * *p* < 0.05, ** *p* < 0.01, *** *p* < 0.001 and **** *p* < 0.0001. One-way analysis of variance (ANOVA) was used to compare the statistical differences among multiple comparisons, while the unpaired Student’s t-test was used for comparison between two means. The log-rank test was conducted to create survival curves. GraphPad Prism 8 software was used to conduct all the statistical analyses.

## 3. Results

### 3.1. Antiviral Effect of the Candidate Compounds on Influenza Virus Strains

We ultimately obtained 13 candidate compounds, with oseltamivir acid used as a positive control (Supplemental Table S1), to evaluate their in vitro antiviral capacities. A2CA exhibited anti-influenza efficacy on all four tested influenza strains without cytotoxicity (Figure 1B–E, Supplemental Table S1 and Supplemental Figure S1). Interestingly, A2CA showed the ability to combat HIN1 A/ZX/1109, which is known as the oseltamivir-resistant strain, suggesting its potential in dealing with drug resistance issues.

### 3.2. A2CA Treatment Reduces IAV Yield Dose-Dependently

**Figure 2:**
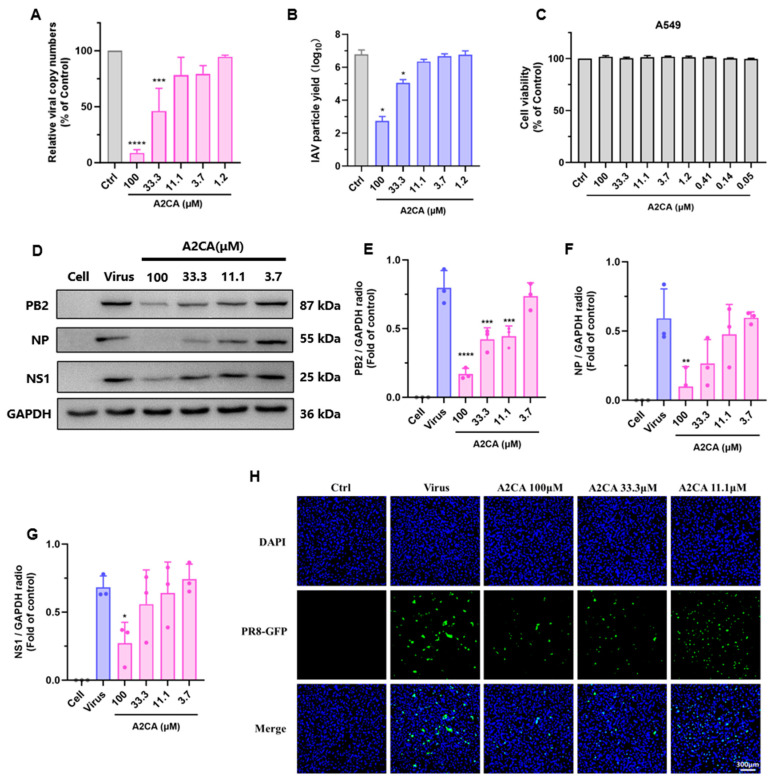
In vitro inhibitory effect of A2CA on H1N1 PR/8. (**A**,**B**) Viral RNA reduction assay and IAV particle yields. MDCK cells were treated with H1N1 PR/8 (MOI = 0.01) with different concentrations of A2CA. After 24 h, the yield of virus particles and cellular RNA load were determined using a PFU assay and a qRT−PCR assay. (**C**) Cell viability of A549 cells after A2CA treatment. (**D**) Inhibitory effects of A2CA on viral PB2, NP and NS1 protein expression. A549 cells were treated with the H1N1 PR/8 strain (MOI = 0.01) in the presence of different concentrations of A2CA. At 24 hpi, the total protein was obtained for Western blotting analysis. (**E**–**G**) Protein levels of PB2, NP and NS1 were quantified by ImageJ software (version 1.53a). (**H**) Fluorescence imaging of A549 cells treated with the H1N1 PR/8-NS1-GFP reporter virus and A2CA treatment. A549 cells were infected with the H1N1 PR/8-NS1-GFP reporter virus (MOI = 0.1) in the presence of A2CA. At 16 h post infection (hpi), the cells were fixed for 2 h and subjected to immunofluorescence assays. The cells were stained with DAPI (blue), and the PR/8-NS1-GFP virus is shown in green. Scale bar: 300 µm. * *p* < 0.05, ** *p* < 0.01, *** *p* < 0.001 and **** *p* < 0.0001.

To further examine whether A2CA inhibits viral infections, we conducted a virus yield reduction assay. Firstly, qRT-PCR and PFU assays were conducted to investigate the numbers of viral RNA load in the MDCK cells and viral particles in the supernatant, respectively. As expected, A2CA observably decreased the viral RNA copies in cells and the infectious viral particles in the supernatant dose-dependently (Figure 2A,B), as compared with the positive control favipiravir (Supplemental Figure S2A). Then, we determined the safety of A2CA on A549 cells at tested concentrations (Figure 2C and Supplemental Figure S1). At safe concentrations ranges, we further performed fluorescence and Western blot assays to determine the viral proteins in A549 cells incubated with the H1N1 PR/8-NS1-GFP reporter virus or the PR/8 strain in the presence of A2CA. As shown in Figure 2D–H, A2CA dose-dependently suppressed viral protein levels.

### 3.3. In Vivo Anti-IAV Activity of A2CA

**Figure 3:**
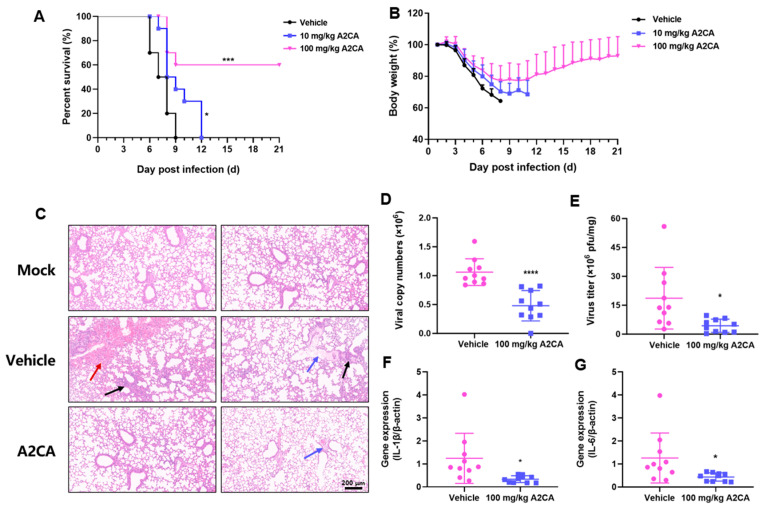
A2CA exerts in vivo anti-IAV effects against H1N1 PR/8 infection**.** Three groups of BALB/c mice (aged 7 weeks, female, *n* = 10 for each group) were intragastrically administered with A2CA or vehicle at 10 mg/kg and 100 mg/kg for 5 consecutive days. After the third day of administration, each mouse received an infection intranasally with 3LD_50_ of the H1N1 PR/8 strain. Survival (**A**) and body weight (**B**) were recorded every day until 21 dpi. Similar experimental procedures were applied for further pathological examination (**C**), influenza RNA copy number determination (**D**), infectious particle detection (**E**) and IL-6 (**F**) and IL-1β (**G**) detection in lung tissues in mice that were sacrificed at 2 dpi. * *p* < 0.05, *** *p* < 0.001 and **** *p* < 0.0001.

Considering the antiviral effect of A2CA in vitro, we next assessed the antiviral performance of A2CA in a lethal infection animal model. As shown in Figure 3A,B, all vehicle-treated mice rapidly lost weight and died within 6 to 9 days post infection (dpi), while a 100 mg/kg A2CA treatment for five consecutive days caused relatively small weight reduction at the beginning and facilitated a progressive restoration of body weight. Notably, A2CA treatment rescued 60% of the mice from a fatal IAV challenge (*p* < 0.001).

Furthermore, the mice were sacrificed at 2 dpi using experimental procedures similar to those above for further examination of histopathology and lung tissue viral loads and inflammatory factor levels. As shown in Figure 3C, the histopathological assessment of lung damage in necropsied mice revealed vascular congestion (red arrow), edema (blue arrow) and necrosis (black arrow) with inflammatory cell infiltration. However, these features were alleviated in the mice treated with A2CA, demonstrating that A2CA could mitigate lung tissue damage induced by the viral infection. Then, we proceeded to perform qRT-PCR and PFU assays to evaluate the viral loads. Our studies demonstrated that the A2CA treatment remarkably suppressed the production of viral RNA and infectious particles (*p* < 0.0001 or <0.05, Figure 3D,E). In addition, A2CA significantly inhibited the signs of IAV-triggered inflammation, including IL-1βand IL-6 (*p* < 0.05, Figure 3F,G). This evidence supports A2CA as a potential inflammatory immune modulator that combats influenza virus infections.

### 3.4. RIG-I/STAT1 Signaling Pathway Activation Induced by IAV Disappeared After A2CA Treatment

**Figure 4:**
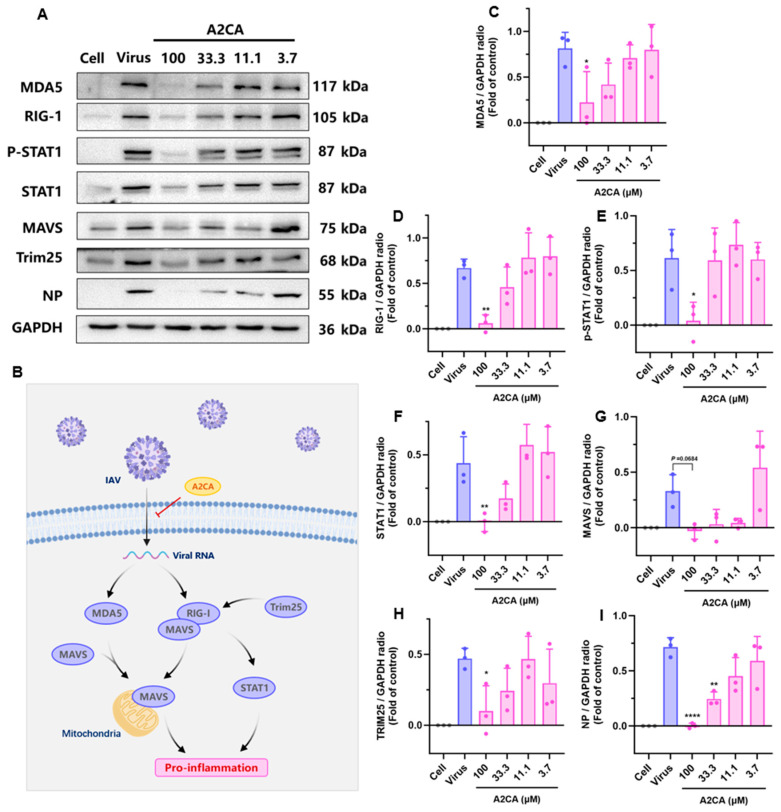
IAV-induced activation of the RIG-I/STAT1 signaling pathway was suppressed after A2CA treatment. (**A**) Western blot analysis for MDA5, RIG-I, p-STAT1, STAT1, MAVS, TRIM25 and NP in A549 cells. (**B**) Schematic diagram of A2CA-mediated downregulation of proteins upregulated by IAV. (**C**–**I**) The quantitative analysis of the proteins was done using ImageJ. A549 cells were treated with the H1N1 PR/8 strain (MOI = 0.01) and A2CA. At 24 hpi, the total protein was obtained for Western blot analysis. Statistical significance was calculated using one-way ANOVA. * *p* < 0.05, ** *p* < 0.01, and **** *p* < 0.0001.

The RIG-I/STAT1 signaling pathway is involved in the inflammatory response and immune disorders, and can be activated during an IAV infection. Compared with those in the cell control group, the protein levels of melanoma differentiation-associated gene 5 (MDA5), RIG-I, p-STAT and tripartite motif-containing protein 25 (TRIM25) in the virus group was remarkably enhanced (Figure 4). Interestingly, A2CA treatment markedly decreased the expression of these proteins in a dose-dependent manner. Although the increase of mitochondrial antiviral signaling protein (MAVS) was not statistically significant, we still observed obvious downregulation after A2CA treatment. Together, these data reveal that the activation of the RIG-I/STAT1 signaling pathway triggered by the IAV did not appear after the A2CA treatment.

**Figure 5:**
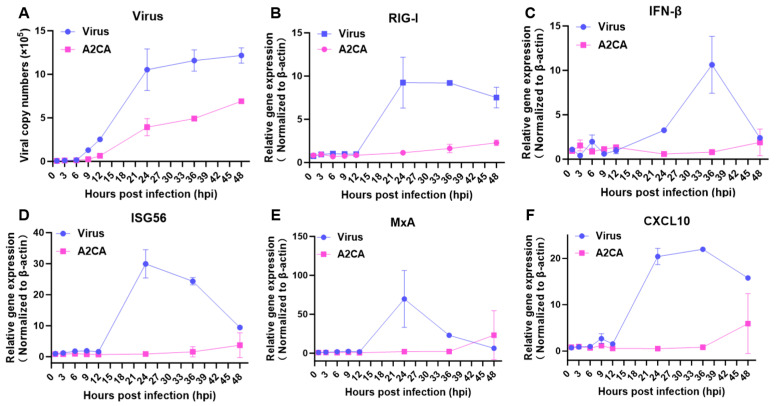
A2CA treatment reduced the yield of IAV and caused almost no alterations in the immune response. A549 cells were treated with the H1N1 PR/8 strain (MOI = 0.01) in the presence or absence of A2CA. Total cellular RNA at different time points was obtained, and the qRT−PCR assay was used to detect the viral RNA copies and the levels of factors associated with the immune response. (**A**) Viral curve. (**B**–**F**) Immune curve.

Additionally, we further examined the immune alterations triggered by the IAV infection at different time points. Our results demonstrated that the IAV infection caused increases in the levels of immune factors, including RIG-I, IFN-β, ISG56, MxA and CXCL10, which peaked from 24 to 36 hpi (Figure 5). In contrast, the A2CA treatment suppressed the yields of the IAV and caused almost no alterations in the above immune factors. These results suggest that A2CA may act directly on the influenza virus itself or others.

### 3.5. A2CA Affects the Viral Replication Stage

**Figure 6:**
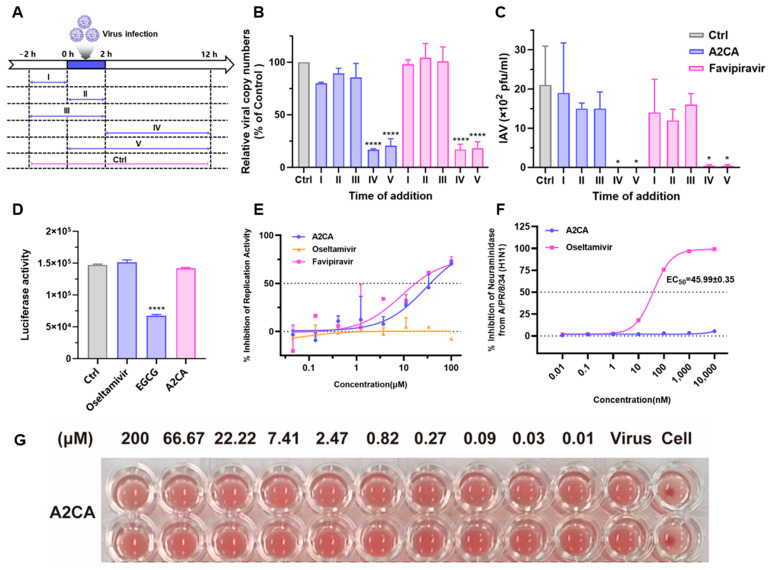
A2CA effects at the IAV replication stage. (**A**) Different treatment of the time-of drug-addition assay. The cells were treated with the influenza H1N1 PR/8 strain at 0 h and 2 h. The addition of A2CA (100 μM) was divided into five phases (I–V). (**B**) Viral RNA copy numbers in cell lysates were determined by qRT-PCR. (**C**) Viral titers of influenza virus in the supernatant were tested through a PFU assay. (**D**) Influenza pseudovirus assay. MDCK cells were infected with influenza pseudotype particles in the presence of A2CA, oseltamivir acid (negative control) or EGCG (positive control). (**E**) Replicon assay. The positive control was favipiravir, while the negative control was oseltamivir acid. (**F**) Neuraminidase inhibition assay. The positive control was oseltamivir acid. (**G**) Hemagglutination inhibition. The positive control was PBS without the virus, while the negative control was the virus alone. * *p* < 0.05, and **** *p* < 0.0001.

We then try to clarify the mechanism of A2CA. Firstly, a time-of-addition assay was carried out (Figure 6A). The qRT-PCR and PFU assay results suggested that A2CA was only effective in phases IV and V (IAV production and viral RNA replication), which means that it was not an inhibitor of viral attachment and entry (*p* < 0.0001 or <0.05, Figure 6B,C). Simultaneously, the influenza pseudovirus and replicon assay results also provided similar evidence to validate that A2CA was an influenza virus replication inhibitor like favipiravir (Figure 6D,E). Additionally, neuraminidase inhibition (NI) and hemagglutination inhibition (HI) assays showed that A2CA did not influence neuraminidase activity and the entry of influenza viruses (Figure 6F,G).

**Figure 7:**
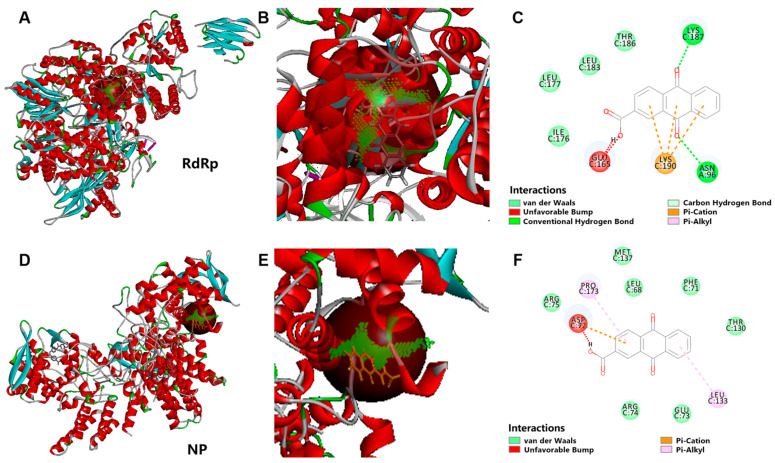
The molecular docking analysis of the RdRp protein and NP with A2CA. (**A**–**C**) Molecular docking of IAV H1N1 RdRp with A2CA. (**D**–**F**) Molecular docking of IAV H1N1 NP with A2CA.

The influenza virus RdRp protein and NP are key enzymes in its genome replication. We further carried out a molecular docking analysis of the IAV RdRp protein and NP with A2CA, respectively. The results showed that the RdRp protein had 58 binding sites, 13 of which were A2CA-binding sites, with four of those sites being potentially stable binding sites (binding fraction was greater than 35). Meanwhile, the NP had 64 binding sites, eight of which were A2CA-binding sites, with four of those sites being potentially stable binding sites (binding fraction was greater than 35). The binding energies of the eight sites were further investigated, and it was revealed that stable binding could occur at these eight sites. The molecular docking analysis of the RdRp protein and A2CA is displayed in Figure 7A–C (RdRp protein) and 7D,E (NP). Collectively, these data indicate that A2CA affects the viral replication stage and may target the IAV RdRp protein and NP. Which specific protein does A2CA directly act on still needs further verification.

### 3.6. Transcriptome Analysis

**Figure 8:**
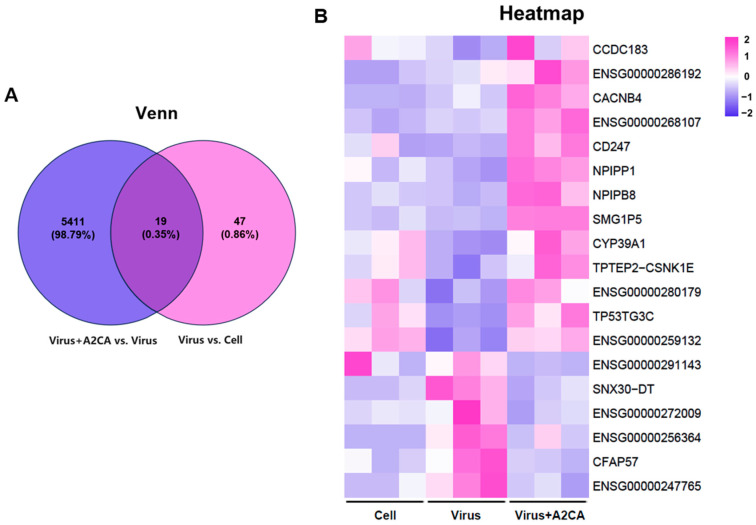
Transcriptome analysis of infected cells after A2CA treatment. DEGs between infected cells with and without A2CA treatment at 18 hpi were obtained by the transcriptome analysis. (**A**) Venn diagram. (**B**) Heatmap.

To investigate the potential molecular alterations responsible for this antiviral activity, we examined the transcriptome analysis of A2CA treatment against IAV infection. As depicted in Figure 8A, the differentially expressed genes (DEGs) among the different groups was determined by Venn analysis. As a result, 19 DEGs were identified. The related DEGs are presented in a heatmap (Figure 8B). Among these DEGs, TP53-target gene 3 protein (TP53TG3C), cilia and flagella associated protein 57 (CFAP57) and sorting nexin 30 divergent transcript (SNX30-DT) may be involved in the antiviral effects of A2CA. As we know, TP53 can be activated when cells are subjected to various stresses, including an influenza virus infection, and then induce cell cycle arrest, apoptosis or antiviral gene expression. In addition, TP53 directly stimulates the expression of the immune-responsive genes. Among them, ISG15, Toll-like receptor 3 (TLR3), protein kinase RNA-activated (PKR), IFN regulatory factor 5 (IRF5) and IRF9 play crucial roles in triggering antiviral responses [17]. According to the report, the NS1 protein of influenza virus is able to modulate the alternative splicing of TP53 through the host factor CPSF4, thus affecting p53 activity and antiviral response [18]. As a target gene of TP53 [19], we hypothesized that TP53TG3C may be involved in regulating the host’s immune response against the influenza virus. Additionally, TP53TG3C could serve as a downstream effector of TP53 to assist TP53-induced cell cycle arrest, thereby limiting influenza viruses from using cellular replication mechanisms for their own reproduction. In addition, CFAP57, a conserved part of flagella and motile cilia, has been proven to form complex connecting components that are vital for motility [20,21]. It has been reported that mutations in the CFAP57 protein lead to primary ciliary dysmokinesis [22]. In the respiratory tract, the normal motility of cilia plays a key role in removing pathogens, mucus, etc. from the respiratory tract [21]. If CFAP57 is dysfunctional, it may lead to ciliary structural or functional defects, which reduces the ability of the respiratory tract to clear pathogens, thereby increasing the chance of pathogens such as influenza viruses entering and infecting the respiratory tract. Additionally, there is a reported possible link between CFAP57 and SARS-CoV-2 through the phosphoproteome [23]. Finally, SNXs are for the transportation of cargoes from endosomes to either the plasma membrane or the trans-Golgi network. SNX5 and SNX27, known as the key components of SNXs, can influence several stages of the viral life cycle (facilitating the entry of viruses into cells, participating in viral replication and promoting the assembly of virions) [24]. SNX30-DT, as a member of the SNXs family, may have a similar function. Although there is no direct evidence to prove a link between the above factors and influenza virus, these findings offer an understanding of the mechanism beneath the anti-influenza effects of A2CA and present new paths for the hunt of creative anti-influenza targets.

## 4. Discussion

Acute lung injury induced by an influenza virus infection is a type of critical and severe disease with a high progression rate and significant lethal risk. It often involves overactive host immune responses that are the main cause of the high mortality rate of influenza [25,26]. The main pathological features are diffuse alveolar damage, pronounced pulmonary edema, large amounts of protein-rich exudate and excessive inflammation, which give rise to chest pain, severe pneumonia, high fever, cough, pharyngitis, chills, gastrointestinal symptoms and, in serious cases, acute respiratory distress syndrome (ARDS) [2,27,28]. A prospective nationwide surveillance conducted in China from 2009 to 2019 on patients of all age with acute respiratory infections demonstrated that influenza virus made up 28.5% of the overall cases [29]. Influenza infection poses great threats to public health and causes enormous financial losses.

Despite advances in the treatment of influenza infection, several concerns remain. Influenza virus, a negative-sense and single-stranded RNA virus, is more prone to undergoing genetic mutation and reassortment. Such frequent mutation and reassortment of influenza viruses make the influenza vaccine outdated by the upcoming flu season [30], and healthcare providers suggest using anti-influenza drugs. However, emerging flu variants have rapidly acquired resistance to the currently employed first-line virus-targeting agents, such as NA inhibitors (zanamivir, peramivir and oseltamivir) [13,31]. Additionally, with the rapid emergence of drug-resistant strains shortly after the launch of the next-generation anti-influenza agent baloxavir marboxil [32,33], the discovery of novel agents has become urgent for scientists. In this study, we screened A2CA from the Chinese traditional medicine Bajitian on the basis of its predicted antiviral activity against influenza strains, especially oseltamivir-resistant strains, and verified its efficacy by various experimental means. Notably, the pre-administration of A2CA significantly ameliorated IAV-mediated weight loss, lung injury and cytokine dysregulation and safeguarded mice against a fatal IAV infection in an influenza mouse model.

In vivo research has reported that RIG-I-mediated IFN generation plays a crucial role in modulating susceptibility to IAV and conferring protection against lethal IAV challenge [34,35,36]. The innate immune system possesses a diverse set of pattern recognition receptors (PRRs), which constitute the main defensive barrier against infectious agents [37]. During the replication of influenza viruses, viral RNA with a 5′-triphosphate end is synthesized and subsequently transferred to the cytoplasm [38]. The cytosolic sensor MDA-5 or RIG-I can recognize this pathogen-associated molecule (PAM), which is then ubiquitinated by TRIM25, triggering various cellular signal cascades. These cascades cause transcription factors to bind jointly to particular locations on the IFN-β promoter, thereby starting the synthesis of IFN-β for antiviral purposes [39,40]. We explored whether RIG-I signaling is influenced by A2CA during an IAV infection. Our findings revealed that the expressions of MDA-5, RIG-I and TRIM25 were increased after an IAV infection, and the situation was markedly abrogated when treated with A2CA. Moreover, although we did not detect a significant decrease in MAVS expression, we could still observe a downwards trend after A2CA treatment. It has been reported that IL-6 and IL-1β are remarkably elevated in the bronchoalveolar lavage fluid (BALF) of patients with persistent ARDS [41,42]. Our in vivo results demonstrated that pre-stimulation with A2CA prior to the IAV infection downregulated IL-6, and IL-1β. Previous findings suggest a clear link between STAT1 activation and RIG-I expression. The recognition of viral RNA by RIG-1 eventually triggers the secretion of type I interferon (IFN), which binds to IFN receptors on the cell surface and activates interferon-stimulated response elements (ISRE) by signaling pathways such as JAK-STAT, thus initiating the transcription and expression of several IFN-stimulated genes (ISGs), such as MxA [43,44]. RIG-I knockdown and overexpression experiments have verified that RIG-I is vital for STAT1 activation [45,46]. According to these facts, our findings showed that the augmentation of STAT1 activation through overexpression of RIG-I was decreased after A2CA treatment. The findings revealed that the RIG-I/STAT1 signaling cascades may not be activated during A2CA treatment and A2CA may act directly on the virus itself.

Moreover, we sought to identify the mechanisms by which A2CA displays its antiviral activities. The time-of-addition assay suggested that A2CA affected the last intracellular replication stage, which was verified by an IAV replicon assay. In addition, a series of experiments excluded the activity of A2CA on the NA and HA proteins. The RdRp protein is crucial in its genome replication. We further investigated the binding of the IAV RdRp protein and NP with A2CA via molecular docking. The results showed that A2CA has strong binding affinity to the IAV RdRp protein and NP, and the anti-influenza effect of A2CA may be connected to the targeting of them. Finally, through transcriptome analysis, we identified several genes that may be involved in the anti-influenza effects of A2CA, including TP53TG3C, CFAP57 and SNX30-DT. This study offers valuable comprehensions to the mechanisms underpinning the anti-influenza activity of A2CA. However, it is crucial to note that our current work on the effects of A2CA on influenza remains in an exploratory phase. When relying solely on the limited gene expression changes detected by RNA-seq, we cannot comprehensively determine which specific genes are involved in the antiviral response. Thus far, we have merely put forward several hypotheses regarding potential antiviral target genes. Consequently, in future research, experimental validation will be of utmost importance. Techniques such as gene knockdown or overexpression should be employed to directly demonstrate the impact of these genes on viral replication. This will not only solidify our understanding of the antiviral mechanisms of A2CA but also lay a more robust foundation for further exploration in this area.

In conclusion, this study exhibited that A2CA has antiviral potential. Followed experiments demonstrated that A2CA has a therapeutic impact in dealing with the injury caused by influenza through multiple mechanisms, including acting on viral replication and the regulation of certain genes. These results could provide a novel perspective on the treatment of influenza virus infections.

## Data Availability

All data can be found in the main text and Supplementary Materials.

## Supplementary Materials

The following supporting information can be downloaded at: https://www.mdpi.com/article/10.3390/v17050628/s1, Figure S1: The phase contrast image of A2CA (100 μM) in MDCK cells and A549 cells; Figure S2: The in vivo and in vitro anti-influenza viral activity of favipiravir; Table S1: In vitro antiviral activities of 13 candidate compounds from Morinda officinalis against multiple influenza virus strains; Table S2: The primers and probe sequences; Table S3: The antibody information.
