# Supplementary material for: Anthraquinone-2-Carboxylic Acid Is a Potential Antiviral Candidate Against Influenza Viruses In Vitro and In Vivo
Source: Viruses. 2025 Apr 27;17(5):628. doi: 10.3390/v17050628 (PMC12115614; doi:10.3390/v17050628)
Supplement: Supplementary file 1 [file viruses-17-00628-s001.zip › viruses-3570829-supplementary.pdf]

Supplemental material

Supplemental Table S1. In vitro antiviral activities of 13 candidate compounds from *Morinda officinalis* against multiple influenza virus strains.

		H1N1 A/PR/8/34		H1N1 ZX/1109		H3N2 A/HK/8/68		B B/Lee/40	
	CC_{50}	EC_{50}	SI	EC_{50}	SI	EC_{50}	SI	EC_{50}	SI
Anthraquinone-2-carboxylic acid	> 100	37.11±2.82	> 2.69	33.34±0.33	> 3.00	73.45±14.71	> 1.36	33.59±3.15	> 2.98
1-Hydroxyanthraquinone	> 100	> 100	-	23.07±1.12	> 4.33	> 100	-	> 100	-
Tectoquinone	> 100	> 100	-	48.67±11.29	> 2.05	> 100	-	> 100	-
Rubiadin-1-methyl ether	> 100	> 100	-	12.28±0.18	> 8.14	58.12±1.44	> 1.72	> 100	-
α-Zingiberene	> 100	> 100	-	> 100	-	> 100	-	> 100	-
Oseltamivir	> 100	3.81±0.11	> 26.25	> 100	-	< 0.046	> 2173.91	> 100	-
Ursolic acid	21.72±3.91	> 100	-	> 100	-	> 100	> 100	> 100	-
Asperulosidic acid	> 100	> 100	-	> 100	-	> 100	> 100	> 100	-
Asperuloside	> 100	> 100	-	> 100	-	> 100	> 100	> 100	-
2-Undecanone	> 100	> 100	-	> 100	-	> 100	> 100	> 100	-
Geniposidic Acid	> 100	> 100	-	> 100	-	> 100	> 100	> 100	-
Aucubin	> 100	> 100	-	> 100	-	> 100	> 100	> 100	-
Monotropein	> 100	> 100	-	> 100	-	> 100	> 100	> 100	-

Supplementary table S2. The primers and probe sequences

	Forward primer (5′-3′)	Reverse primer (5′-3′)	Probe (5′-3′)
Influenza H1N1 PR/8	GACCRATCCTGTCACCTCTGAC	AGGGCATTYTGGACAAAKCGTCTA	FAM-TGCAGTCCTCGCTC ACTGGGCACG-BHQ1
Mouse IL-1β	CGTGCTGTCGGACCCATATGAG	GCCCAAGGCCACAGGTATTT	
Mouse IL-6	AGTTGCCTTCTTGGGACTGA	GACGTGGAACTGGCAGAAGAG	
Mous β-actin	TGTCCACCTTCCAGCAGATGT	AGCTCAGTAACAGTCCGCCTAGA	
Human CXCL10	GGTGAGAAGAGATGTCTGAATCC	GTCCATCCTTGGAAGCACTGCA	
Human RIG-I	GCCTTCAGACATGGGACGAA	ACTGCTTTGGCTTGGGATGT	
Human IFN-β	CACTACAGCTCTTTCCATGA	AGCCAGTGCTAGATGAATCT	
Human MxA	GCTACACACCGTGACGGATATGG	CGAGCTGGATTGGAAAGCCC	
Human ISG56	CCTCCTTGGGTTCGTCTACA	GGCTGATATCTGGGTGCCTA	
Human β-actin	CTACCTCATGAAGATCCTCACCGA	TTCTCCTTAATGTCACGCACGATT	

Supplementary table S3. The antibody information

Antibody	Dilution	Manufacturer	Cat. No.
Rabbit Anti-PB2	1:1000	Gene Tex	GTX125926
Rabbit Anti-NP	1:1000	Abcam	Ab128193
Rabbit Anti-NS1	1:1000	Gene Tex	GTX125990
Rabbit Anti-TRIM25	1:1000	Proteintech	12573-1-AP
Rabbit Anti-RIG-1	1:1000	Proteintech	20566-1-AP
Rabbit Anti-MDA5	1:2000	Proteintech	21775-1-AP
Rabbit Anti-MAVS	1:2000	Proteintech	14341-1-AP
Rabbit Anti-STAT1	1:1000	Cell Signal Technology	9172T
Rabbit Anti-pSTAT1	1:1000	Cell Signal Technology	9167T
Mouse Anti-GAPDH	1:5000	Abcam	ab8245
